# The Factory Nurse's Salary

**Published:** 1919-03-15

**Authors:** Constance U. Kerr

**Affiliations:** Irlam, near Manchester


*To the Editor of* The Hospital.

Yours faithfully,

G. M. E. L.

### THE FACTORY NURSE'S SALARY.

*To the Editor of* The Hospital.

Sir,—I regret that your correspondent signing herself as "Shell Factory Nurse" is so much disappointed with the remarks regarding salary in my recent article on Factory Nursing.

She must bear in mind that £100 per annum is only quoted by me as an *initial* salary, and that this rises progressively and at a much faster rate than in private and hospital work. Compared with other branches of nursing the work is well-paid. The Health Visitor—even with the additional advantage of the C.M.B. certificate—seldom starts with as much as £100, and the same holds true for School Nurses and other branches of the profession. In none of these cases am I alluding to *bonus*, which in the case of factory nursing is often very considerable, and, in addition, as I have already pointed out, meals are often provided in the factory canteen free. Moreover, the nurse is gaining a training in a new profession—for as I have said in my article, factory nursing is often a stepping-stone to welfare supervision.

As a welfare supervisor of many years standing, I cannot agree with "Shell Factory Nurse" that a factory nurse should receive as much as an assistant welfare supervisor. The nurse is a specialist in *one* branch only—at all events when she first enters the factory—whilst the assistant welfare supervisor is a specialist in *many*. It may have been true that at the beginning of the war, when the demand for welfare workers much exceeded the supply, that "a woman of good education who is well recommended can get an assistant welfare supervisor's post at a salary of £130 to £150," but it does not hold good now. The training is rapidly becoming standardised, and only fully-qualified persons will eventually be appointed. The following are a few of the necessary qualifications for an assistant welfare supervisor:—

(1) A University Degree or its equivalent in educational work.

(2) A knowledge of social or settlement work, and, if possible, a definite training for welfare work on the new lines laid down by the Association.

(3) Knowledge of hygiene and nursing (very often the assistant welfare supervisor is already a trained nurse).

(4) Ability to organise games, classes, etc., is very often required.

(5) A knowledge of canteen work and food values.

(6) Ability to present reports, address meetings., etc., is very necessary.

These are only a *few* of the essentials required by the modern assistant welfare supervisor, so that I do not think the trained nurse can expect to be as well paid, unless she is willing and able to take on, in addition to being responsible for the ambulance room, one or more of the above duties. If she is able to do so, she can expect the increased pay.—Yours faithfully,

Constance U. Kerr, LL.A. (Hons.).

Irlam, near Manchester, March 7, 1919.

